# Effect of *Pseudomonas aeruginosa* Elastase B on Level and Activity of Immune Proteins/Peptides of *Galleria mellonella* Hemolymph

**DOI:** 10.1673/031.012.8801

**Published:** 2012-07-26

**Authors:** Mariola Andrejko, Magdalena Mizerska-Dudka

**Affiliations:** Department of Invertebrate Immunology, Institute of Biology, Maria Curie-Skłodowska University, Akademicka 19, 20-033 Lublin, Poland

**Keywords:** antimicrobial peptides, apoLp-III, lysozyme

## Abstract

Susceptibility of proteins and peptides present in immune hemolymph of *Galleria mellonella* Fabricius (Lepidoptera: Pyralidae) larvae to proteolytic degradation by purified elastase B of *Pseudomonas aeruginosa* was studied. Results showed that apoLp-III protein was gradually digested by elastase B *in vitro.* Additionally, polipeptides with molecular mass 6.5 and 4 kDa were degraded after treatment with the studied enzyme. The lack of these peptides and the decrease in *anti-Escherichia coli* activity could indicate that inducible antimicrobial peptides were digested by elastase B. On the contrary, no change in the lysosome activity level was observed in immune hemolymph incubated with elastase B. Thus, elastase B might contribute to the pathogenesis of *P. aeruginosa.*

## Introduction

The gram-negative bacterium *Pseudomonas aeruginosa* is an opportunistic human pathogen responsible for many types of infectious diseases. Different strains of *P. aeruginosa* secrete several extracellular proteolytic enzymes that have been implicated as virulence factors, namely protease IV, alkaline protease, elastase A, and elastase B (Caballero et al. 2001).

*Pseudomonas aeruginosa* elastase B is one of the major proteins secreted into the environment by many strains of this opportunistic pathogen. This 33 kDa enzyme (also called LasB protease and pseudolysin) belongs to the thermolysin family of Zndependent neutral metalloendopeptidases (M4) (Morihara et al. 1965; Morihara 1995; Kessler et al. 1998). It has a broad specificity, hydrolyzing internal peptide bonds of proteins and peptides on the amino side of hydrophobic residues in position P1’ (Matthews 1988; Miyoshi and Shinoda 2000). The primary structure of elastase was deduced from a full nucleotide sequence (Bever and Iglewski 1988; Fukushima et al. 1989), and its three-dimensional structure was determined by Thayer et al. (1991). Elastase B is involved in pathogenesis by degradation of human immunologically competent particles. LasB destroys complement components (Schultz and Miller 1974), cytokines (Parmely et al. 1990), immunoglobulins IgA and IgG (Buret and Cripps 1993; Maeda and Yamamoto 1996), human airway lysozyme (Jacquot et al. 1985), proteinase-activated receptors (Dulon et al. 2005), and surfactant protein A and D (Mariencheck et al. 2003).

Insects have a defense mechanisms consisting of cellular and humoral immune response systems (Lavine and Strand 2002; Jiravanichpaisal et al. 2006). The cellular response comprises phagocytosis, encapsulation, and nodulation of non-self bodies. The humoral defense involves production of antimicrobial peptides, reactive oxygen and nitrogen intermediates and complex enzymatic cascades that regulate coagulation and melanization of hemolymph (Lavine and Strand 2002). Antibacterial peptides are mainly produced in the fat body or hemocytes and then released into the hemolymph. Their synthesis is induced (i.e., cecropins, attacins, etc.) or increased (lysozyme) in response to foreign entities (Bulet et al. 1999; Yu et al. 2002 ).

It has been shown that apolipophorin III, a major exchangeable lipid transport protein found in hemolymph, may play an important role in the insect immune response. Recent immune studies indicate that apoLp-III stimulates an increase in hemolymph antibacterial activity (Wiesner et al. 1997; Niere et al. 1999) and may act as a pattern recognition molecule (Dettlof and Wiesner 1999; Whitten et al. 2004). ApoLp-III enhances hemocyte phagocytosis activity (Wiesner et al. 1997) and stimulates cellular encapsulation of foreign material (Whitten et al. 2004).

Andrejko et al. (2005) indicated that proteases IV might be involved in *P. aeruginosa* pathogenesis by degradation of *G. mellonella* apoLp-III. On the other hand, another immune protein, lysozyme, seemed to be insensitive to this protease (Andrejko et al. 2005). This raised questions on whether another *P. aeruginosa* protease, elastase B, is engaged in pathogenesis. This paper presents *in vitro* studies on the effect of purified elastase B of *P. aeruginosa* on the activity and level of proteins and peptides in the immune hemolymph of *Galleria mellonella* Fabricius (Lepidoptera: Pyralidae) larvae.

## Materials and Methods

### Insect culture and immune challenge

Larvae of the greater wax moth *G. mellonella* were reared on a natural diet of honeybee nest debris at 30 °C in the dark. Final instar larvae weighing 250–300 mg were selected for this study.

The larvae were immune-challenged by an injection of live *Escherichia coli* D31 (10^5^ CFU). After the treatment, larvae were kept at 30 °C in the dark on sterile Petri plates, and hemolymph was collected after 24 hours.

### Bacteria and enzyme

*Escherichia coli* K12, strain D31, LPS defective, streptomycin and ampicillin resistant (CGSC 5165) was used (Boman et al. 1974). The bacterial cells were grown in a nutrient broth for 24 hours at 37 °C and pelleted by centrifugation at 20,000 × g for 10 min at 4 °C.

Purified, crystallized elastase B of *P. aeruginosa* was purchased from Calbiochem (www.emdmillipore.com).

### *In vivo* experiments

For *in vivo* experiments, *G. mellonella* larvae were injected with elastase B at concentrations of 0.05 µg, 0.1 µg, and 0.2 µg per larvae. Groups of 12 larvae were used in each case. After challenge, insects were kept on sterile Petri plates at room temperature in the darkness. The percent mortality of larvae 48 hours after enzyme injection was determined.

### Hemolymph collection and preparation of hemolymph extract

Prior to hemolymph collection, the insects were chilled for 15 min at 4 °C. Hemolymph samples were obtained by puncturing the larval abdomen with a sterile needle. The outflowing hemolymph was immediately transferred into sterile and chilled Eppendorf tubes containing a few crystals of phenylthiourea (PTU) to prevent melanization. The hemocyte-free hemolymph was obtained by centrifugation at 200 × g for five min and subsequently at 20,000 × g for 10 min at 4 °C. Pooled supernatants were stored at -20 °C until used.

Low molecular mass proteins and peptides were isolated from the hemocyte-free hemolymph by the acidic/methanol extraction method adapted from Schoofs et al. (1990). The hemolymph was diluted 10 times with the extraction solution consisting of methanol: glacial acetic acid: water (90:1:9, v/v/v) and mixed thoroughly. Precipitated proteins were pelleted by centrifugation at 20,000 × g for 30 min at 4 °C. The obtained supernatant was collected, vacuum dried, and the pellet was stored at -20 °C until used. Before used, it was dissolved in an appropriate volume of sterile distilled water. The acidic/methanol hemolymph extract obtained as described above contained proteins and peptides of M_r_ below 30 kDa.

### Antibacterial activity assay

The presence of antibacterial activity in the hemolymph and acidic/methanol extracts of hemolymph was detected by a growth inhibition zone assay using solid agar plates containing viable *E. coli* cells as described by Hoffmann et al. (1981). To improve the sensitivity of the method, chicken egg white lysozyme (EWL) at a concentration of 2.0 mg × mL^-1^ of the medium was added (Chalk and Suliaman 1998; Cytryńska et al. 2001). Each well on the Petri dish was filled with 4 µL of hemolymph or hemolymph extract (the amount of protein and other reaction details are presented in Figure legends). The agar plates were then incubated at 37 °C for 24 hours. The diameters of *E. coli* D31 growth inhibition zones were measured and the level of antimicrobial activity was calculated using the algorithm described by Hultmark et al. (1982). Synthetic cecropin B (Sigma-Aldrich, www.sigmaaldrich.com) was used as a standard for evaluation of antibacterial activity

### Lysozyme activity

Lysozyme activity in hemocyte-free hemolymph and in hemocyte-free hemolymph extract was detected using agarose plates containing freeze-dried *Micrococcus luteus* (Sigma) (Jarosz 1995). Each well on the Petri dish was filled with 4 µL sample, and after 24 hours incubation at 28 °C, the diameters of the *M. luteus* lytic zones were measured. The activity of lysozyme was calculated from a standard curve made with EWL (Sigma; EC 3.2.1.17) and expressed in µg/mL EWL.

### Electrophoresis methods and immunoblotting

Polyacrylamide gel electrophoresis of protein samples was performed by tricine SDS-PAGE (16.5% T, 3% C) according to Schägger and Jagow (1987). Polypeptide bands in gels were stained with Coomassie Brilliant Blue G-250 (Imperial Chemical Industries).

IEF was performed with 100 µg of hemolymph extract protein using the Protean IEF focusing system (Bio-Rad, www.biorad.com) according to the manufacturer's recommendations. The sample was suspended in rehydratation buffer (8.8 mol/L urea, 2%, W/V CHAPS; 70 mmol/L DTT; 0.2 %, W/V, Bio-Lytes) and loaded on 70 mm IPG strips (Bio-Rad). After separation of proteins in the first dimension, strips were equilibrated twice for 15 min in equilibration buffer (6 mol/L urea; 20%, V/V, glycerol; 2%, W/V, SDS; 375 mmol/L Tris-HCL, pH 8.8). The first step was done in equilibration buffer with 130 mmol/L DTT; the second equilibration step contained 135 mmol/L iodoacetamide. Then tricine SDS-PAGE and immunoblotting were performed under the conditions described above.

The samples after tricine and 2D electrophoresis were electroblotted onto Immobilon membranes (Millipore, www.millipore.com) for 90 min at 350 mA. For identification of apolipophorin-III, anti*G mellonella* apoLp-III antibodies (Agrisera, www.agrisera.com) were used. Alkaline phosphatase-conjugated goat anti-rabbit IgGs were used as secondary antibodies. Immunoreactive bands were visualized by incubation with p-nitroblue tetrazolium chloride and 5-bromo-4-chloro-3-indolyl phosphate.

### Protein determination

The protein concentration was estimated by the Bradford (1976) method using bovine serum albumin (BSA) as a standard.

## Results

### Mortality studies

**Figure 1.**
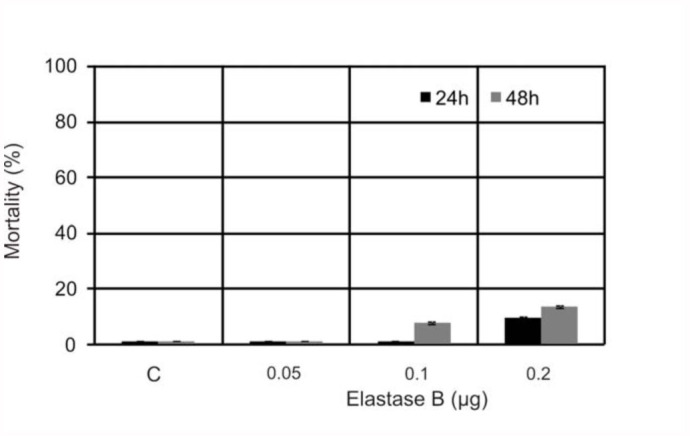
The toxicity of *Pseudomonas aeruginosa* elastase B to *Galleria mellonella* larvae. Mortality percentage of insects after injection with indicated elastase B concentrations at 24 (black) and 48 (gray) hours. (C) Control, punctured only larvae. All values represent the mean ± SD of three independent experiments. High quality figures are available online.

First, the toxicity of *P. aeruginosa* elastase B up to the 7^th^ instar larvae of *G. mellonella* was determined (Figure 1). Three doses of purified elastase B (0.05, 0.1, and 0.2 µg/larvae) were used. Insects were injected through the last pro-leg and mortality rates were determined over a 48 hour period. The mortality rates observed for larvae inoculated with elastase at the concentration of 0.1 and 0.2 µg per larvae after 48 hours incubation were 8 and 14%, respectively. Results indicated that the doses of purified elastase B were sublethal for *G. mellonella* larvae and these doses were chosen for further experiments.

### Effect of elastase B on hemolymph proteins/peptides profile

Subsequent experiments investigated the susceptibility of proteins and peptides present in immune hemolymph of *G. mellonella* larvae to proteolytic degradation by purified elastase B of *P. aeruginosa.* Acidic/methanol extract obtained by the method adopted from Schoofs et al. (1990) was used as the source of proteins and peptides. This procedure allowed elimination of hemolymph proteins of molecular masses above 30 kDa (Cytryńska et al. 2007).

**Figure 2.**
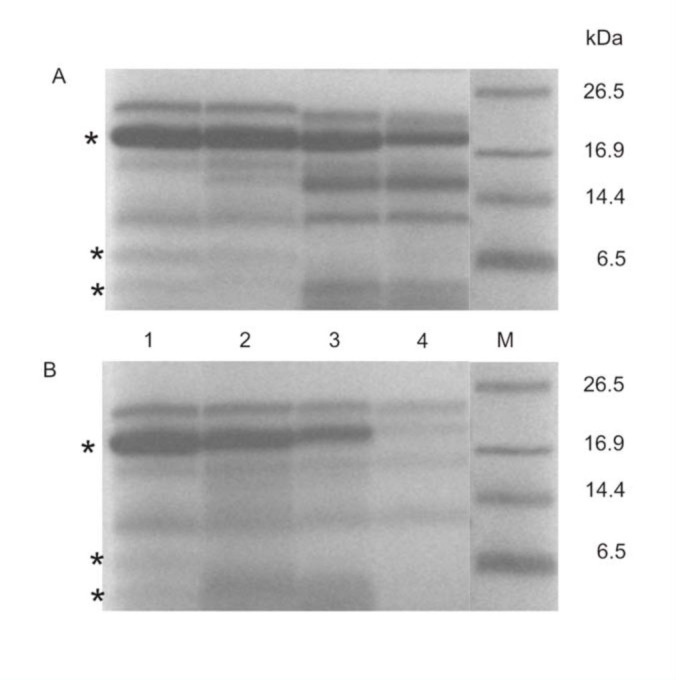
The effect of elastase B on immune hemolymph extract of *Galleria mellonella* larvae *in vitro.* Samples containing immune hemolymph extract (20 µg of protein) and purified elastase B (0.05 µg (lane 2), 0.1 µg (lane 3), 0.2 µg (lane 4)) were incubated at 37 °C for 60 min. Then samples were separated by tricine electrophoresis and stained with Commassie Brilliant Blue R-250. (M) Molecular mass markers. Control (hemolymph extract only) in lane 1. (*) Indicates apoLp-III (18 kDa) and antimicrobial peptides (6.5 and 4 kDa). High quality figures are available online.

In preliminary studies, the immune hemolymph extract was incubated 60 min at 37 °C with various concentrations of elastase B at 37 °C (optimal for the enzyme) or 30 °C (optimal for the insect). Control extracts were also incubated at both temperatures. All samples were analyzed by Tricine-SDS-PAGE. Polypeptides of molecular mass below 30 kDa had different susceptibility to proteolytic digestion by *P. aeruginosa* elastase B (Figure 2). The results also showed that heat treatment of the control sample did not result in degradation of insect proteins.

### Identification of apoLp-III as a substrate for elastase B

The major protein band of approximately 18 kDa was effectively digested by the elastase B (Figure 2). It corresponded to the molecular mass of apolipophorin III, which is one of the most abundant polypeptides in *G. mellonella* hemolymph. Because apoLp-III participates in the induced immunological responses of the larvae of *G. mellonella,* the question of whether elastase B is responsible for apoLpIII degradation in wax moth hemolymph was addressed.

**Figure 3.**
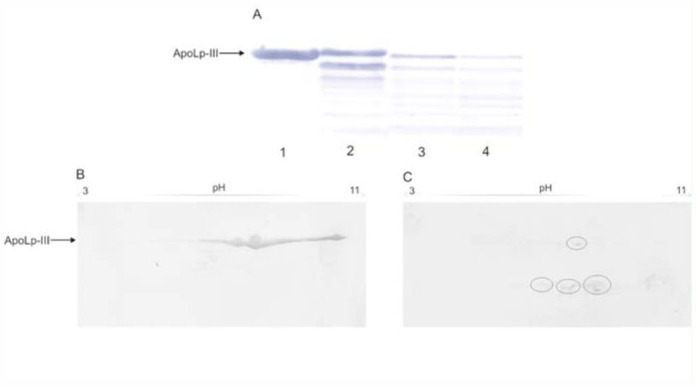
Degradation of apoLp-III from hemolymph extract of *Galleria mellonella* larvae by elastase B *Pseudomonas aeruginosa in vitro.* (A) Tricine SDS-PAGE and immunobloting with anti-*Galleria* *mellonella* apoLp-III antibodies. The samples containing hemolymph extract (20 µg of protein) and elastase B (0.05 µg (lane 2), 0.1 µg (lane 3), 0.2 µg (lane 4), 0.4 µg (lane 5)) were incubated at 37 °C for 60 min. The reactions were stopped by sample buffer addition. Lane 1 contained hemolymph extract only. (B) and (C) SDS-PAGE 2D electrophoresis. Hemolymph extract ( 100 µg of protein) and elastase B (0.6 µg) were incubated at 37 °C for 60 min. Then samples were loaded on pH 3 to l l isoelectric strips followed by Tricine SDS-polyacrylamide gel electrophoresis. For identification of apoLp-III anti-Golleria *mellonella* apoLp-III antibodies were used. (B) control sample of apoLp-III. (C) degradation products of apoLp-III are marked in circles. High quality figures are available online.

ApoLp-III protein was almost completely degraded after the incubation of the hemolymph extract with elastase B used at the concentration of 0.2 µg, particularly after incubation at 37 °C (Figure 2B). Therefore, subsequent experiments tested the apoLp-III level using the electrophoretic/immunoblotting technique. The samples containing the hemolymph extract and elastase B (0.05, 0.1, and 0.2 µg/larva) were incubated at 37 °C for 60 min. Next, the samples after tricine SDS-PAGE were electroblotted onto Immobilon membranes. Anti-G. *mellonella* apoLp-III antibodies were used for identification of apoLp-III. The results of the kinetic studies (Figure 3) revealed that apoLp-III was stepwise degraded to lower molecular mass forms. The amount of apoLp-III protein decreased with the growing concentration of the enzyme. Finally, elastase at the concentration 0.2 µg completely digested apoLp-III protein after 60 min of incubation *in vitro* (Figure 3A, lane 4).

Samples containing hemolymph extract (100 µg protein) preincubated with elastase B (0.6 µg protein) were also tested by two— dimensional gel electrophoresis. IPG strips that separate proteins with pI values of 3 to 11 were used, as well as 16.5% Tris-Tricine-SDS gel. As revealed by immunoblots (Figure 3B), apoLp-III protein is a predominant protein and exists in different isoforms, which M_r_ are indistinguishable. The protein isoforms, however differ in the isoelectric point. As presented in Figure 3C, samples incubated with elastase B showed nearly a complete lack of apoLp-III protein in comparison with the control samples (Figure 3B). The trace amount of apoLp-III was observed together with three degradation products with molecular mass approximately 6 kDa, differing in the isoelectric point. The above results suggest that the decline in the 18 kDa protein level may be caused by proteolytic degradation by *P. aeruginosa* elastase B.

### Identification of antimicrobial peptides as substrates for elastase B

It was found that in the extract of immune hemolymph, at least two peptide bands with molecular mass 4–6 kDa displayed antimicrobial activity (Cytryńska et al. 2007).

Tests were run to see whether antimicrobial peptide activity of *G. mellonella* was abolished by purified elastase B. Samples of cell-free immune hemolymph or hemolymph extract were mixed with *P. aeruginosa* elastase B, and after 60 min of incubation at 37 °C, they were tested for antibacterial activity against the indicator strain *E. coli* D31.

**Figure 4.**
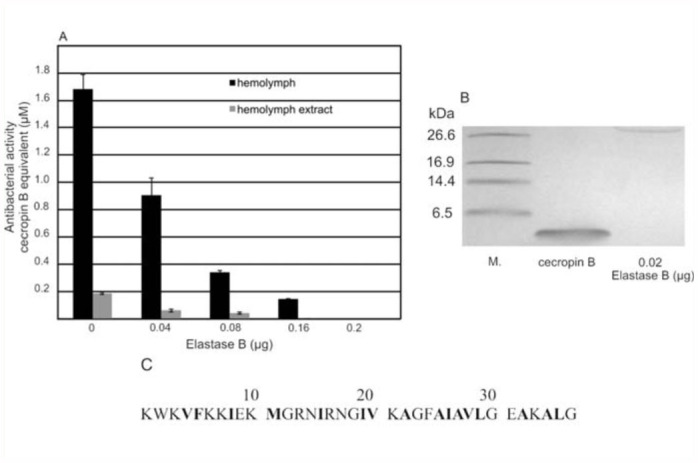
(A) The effect of elastase B on antibacterial activity of *Galleria mellonella* hemolymph. Samples containing immune hemolymph (black) or immune hemolymph extract (grey) (10 µg of protein) and elastase B were incubated at 37 °C for 60 min. Antibacterial activity was analyzed by diffusion well assay as described in the Materials and Methods section. The bars represent ± SD. (B) Degradation *in vitro* of synthetic cecropin B by elastase B. Samples containing synthetic cecropin B (2 µg) and elastase B (0.02 µg 0.04 µg) were incubated at 37 °C for 60 min. The reaction was stopped by sample buffer addition. Then the samples were subjected to tricine-SDS-PAGE. (M) Molecular mass markers. (C) Amino acid sequence of cecropin B *Hyalophora cecropia.* The potential enzyme cleavage sites are indicated in bold. High quality figures are available online.

Figure 4A shows that the antibacterial activity measured by the diffusion well assay was significantly lower in the samples preincubated with *Pseudomonas* elastase. Antibacterial activity of acidic/methanol hemolymph extract was completely abolished by elastase used at the concentration of 0.16 µg. However, in the case of hemolymph, complete inactivation of peptides occurred when elastase was added to the sample at the concentration of 0.2 µg/larvae.

The data presented in Figure 2 indicate that peptide bands with molecular mass approximately 6.5 and 4 kDa were degraded by elastase B, used at the concentration of 0.05 µg/larvae.

The synthetic cecropin B of *Hyalophora cecropia* was used for the comparative study, which showed that cecropin was completely degraded *in vitro* by 0.02 µg of purified elastase B (Figure 4B). The analysis of cecropin B primary structure revealed four potential cleavage sites for elastase B (Figure 4C).

### Studies on lysozyme as a substrate for elastase B

Finally, we investigated whether the level of lysozyme activity changed after treatment with elastase B. It is known that lysozyme plays an important role in humoral defense of *G. mellonella.*

**Figure 5.**
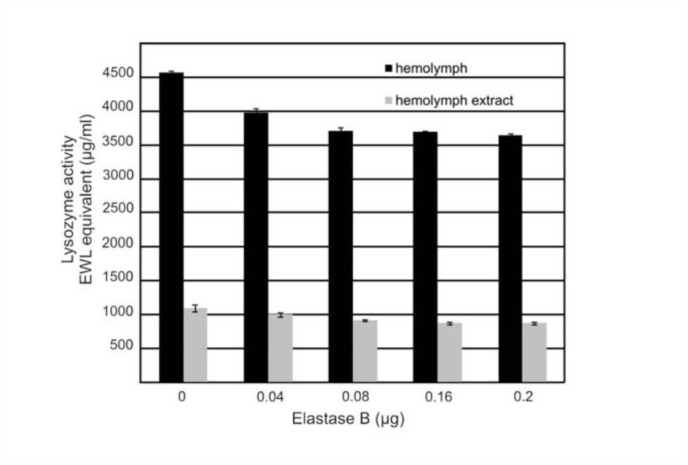
The effect of elastase B on lysozyme activity in *Galleria mellonella.* Samples containing immune hemolymph (black) or immune hemolymph extract (grey) (10 µg of protein) and elastase B were incubated at 37 °C for 60 min. Lysozyme activity in samples was detected by diffusion assay using agarose plates containing freeze-dried *Micrococcus luteus* as described in the Materials and Methods section. High quality figures are available online.

In immune hemolymph and hemolymph extract incubated with elastase B, the activity of lysozyme activity level was reduced (Figure 5). A near 20% decrease in lysozyme activity both in the case of hemolymph and hemolymph extract was observed in samples containing 0.2 µg elastase B. This indicated that, in contrast to apoLp-III and antimicrobial peptides, lysozyme activity appeared to be insensitive to metaloprotease produced by *P. aeruginosa.*

## Discussion

It is known that thermolysin-like metalloproteinases associated with human or entomopathogenic bacteria and fungi play a predominant role as virulence factors. *Pseudomonas aeruginosa* virulence is attributed greatly to its ability to secrete metalloproteases (e.g., elastase B) into the environment. The role of elastase as a virulence factor is supported by inactivation or degradation of a variety of biologically important substrates.

Elastase B has been shown to enhance the virulence of *P. aeruginosa* strains (Morihara and Homma 1985; Nicas and Iglewski 1985). The results of our *in vivo* tests indicated that elastase injected into hemolymph of *G. mellonella* larvae was toxic to insects. The cadavers of killed larvae became black, suggesting activation of prophenoloxidase, which mediates melanization (data not presented).

This study demonstrated that proteins and peptides present in immune hemolymph of *G. mellonella* were susceptible to proteolytic degradation by purified elastase B of *P. aeruginosa.* In particular, elastase B was responsible for degradation of apoLp-III and antimicrobial peptides with molecular mass 4 and 6.5 kDa. Results also showed that *G. mellonella* lysozyme activity appeared to be insensitive to elastase B *in vitro.*

Literature data suggested involvement of apoLp-III in immune response of the greater wax moth (Dunphy and Halwani 1997; Halwani et al. 2000; Pratt and Weers 2004; Whitten et al. 2004). In *G. mellonella,* apoLpIII has a molecular mass of 18.1 kDa, an isoelectric point of 6.5, and 64–90% amino acid sequence homology with apoLp-III of other Lepidopteran species (Weise et al. 1998). Andrejko et al. (2005) showed that apoLp-III was degraded by extracellular serine protease IV isolated from the entomopathogenic strain of *P. aeruginosa in vitro.* The increase in the apoLp-III content *in vivo* was observed during the first 19 hours after injection with *P. aeruginosa,* and a subsequent decrease after prolonged infection time (Andrejko et al. 2008).

It is known that elastase B hydrolyzes internal peptide bonds of proteins on the amino acid side of the hydrophobic residues with phenylalanine, tyrosine, and leucine, which are the preferred residues in position P1’ (Thayer et al. 1991). The analysis of apoLp-III primary structure (Weise et al. 1998) revealed that among 163 amino acid, 23 residues constitute potential cleavage sites for elastase B.

This study showed that the 18 kDa apoLp-III protein was effectively digested by purified elastase B. As revealed by electrophoretic/immunoblotting technique, the apoLp-III was almost completely degraded after the incubation *in vitro* with elastase B used at the concentration of 0.2 µg. This result could indicate that a decreasing level of apoLp-III after prolonged time of infection is connected with degradation of this protein by elastase B.

This seemed to be confirmed by our observation *in vitro* and *in vivo* that the degradation of apoLp-III was correlated with a significant increase of proteolytical activity produced by *P. aeruginosa* during late stages of bacterial infection of the host, *G. mellonella* larvae.

In the extract of immune hemolymph, at least two additional peptide bands with molecular mass 4–6 kDa were detected when compared to the extract prepared from non-immune hemolymph. It was also shown that additional bands displayed antimicrobial activity appearing in the hemolymph in response to immune challenge (Cytryńska et al. 2007). *Galleria mellonella* can release at least 18 antimicrobial peptides from 10 families to defend itself against invading microbes (Brown et al. 2009).

We determined that peptide bands with molecular mass of approximately 6.5 kDa were not observed in the sample treated with 0.05 µg elastase. The defense peptide with M_r_ of 6.5 kDa purified from the hemolymph of immune-challenged *G. mellonella* larvae (Gm anionic peptide 2) is active against certain Gram-positive bacteria and also exhibited antifungal activity (Cytryńska et al. 2007). This could indicate that the studied enzyme was capable of degrading this peptide, causing decreased antimicrobial activity in the hemolymph.

Our results also showed that the peptide band of 4.0 kDa was sensitive to degradation by elastase B. There are at least five different peptides with a molecular mass 4 kDa isolated from immunized *G. mellonella* larvae: Gm anionic peptide, Gm proline-rich peptide 1, defensin, a defensin—like and cecropin D—like peptide (Mak et al. 2001; Lee et al. 2004; Cytryńska et al. 2007).

The decrease in anti—*E. coli* activity, lack of peptide bands with molecular mass 6.5 and 4 kDa, and degradation of synthetic cecropin B indicate that inducible antimicrobial peptides were degraded by the *P. aeruginosa* elastase B.

These results correlate with the studies performed earlier in our laboratory that indicated that elastase B produced by *P. aeruginosa* entomopathogenic strain digested *in vitro* inducible antimicrobial peptides of *G. mellonella* as well as synthetic cecropin B *H. cecropia* (Andrejko et al. 2009).

Lysozyme plays an important role in humoral defense of *G. mellonella.* The lysozyme activity in insect hemolymph is maintained on a low level and increases in response to bacterial challenge (Powning and Davidson 1973; Morishima et al. 1995; Lockey and Ourth 1996). Peptidoglycan and lipopolysacharide fragments released by lysozyme from bacterial cell walls act as signal molecules for the activation of a series of immune genes (Dunn et al. 1985; Kanost et al. 1988;Morishima et al. 1992).

Our earlier studies showed that the level of lysozyme was not degraded *in vitro* by *P. aeruginosa* crude proteolytic fraction (Andrejko 2004) and in the samples containing protease IV (Andrejko et al. 2005). However, a significant decrease in lysozyme activity exposed to the action of *P. aeruginosa* proteins was observed (Andrejko 2004).

The results presented here indicated that after incubation of immune hemolymph with purified elastase, the activity of lysozyme was inhibited by 20% in comparison to control samples. The results are in line with *in vivo* data reported previously (Andrejko et al. 2008). The *G. mellonella* lysozyme level and activity appeared to be insensitive to extracellular proteinases produced during *P. aeruginosa* infection (Andrejko et al. 2008)

Hydrolysis of hemolymph proteins by collagenolytic enzymes, such as thermolysin, results in formation of small-sized protein fragments, inducing expression of immune related genes encoding antimicrobial peptides such as galleriomycin, gloverin, IMPI, and lysozyme (Altincicek and Vilcinskas 2006; Altancicek et al. 2007).

Recently, Andrejko et al. (2011) showed that elastase B injected at a sublethal concentration was responsible for eliciting the humoral immune response in *G. mellonella* larvae. Appearance of antimicrobial peptides, higher level of lysozyme, and apoLp-III were observed in the hemolymph 24 hours after elastase injection, in comparison with control *G. mellonella* larvae. On the other hand, in the present work, both apoLp-III and antimicrobial peptides were substrates for purified elastase B. It seems that in the first hours of *P. aeruginosa* infection, elastase B. stimulates the humoral immune response in *G. mellonella.* Degradation of apoLp-III as with other immune peptides seems to be correlated with a significant increase of proteolytic activity produced by *P. aeruginosa* during late stages of bacterial infection.

The experimental evidence reported here indicates that *in vitro* proteolytic degradation proteins/peptides in larval hemolymph may be caused by elastase B from *P. aeruginosa.* This metalloproteinase of the entomopathogenic strain *P. aeruginosa* seems to contribute to the virulence against insect immune response. Detailed mechanisms by which *P. aeruginosa* elastase B influence *G. mellonella* immune response require further studies.
